# Perceptions and experiences of stigma among parents of children with developmental disorders in Ethiopia: A qualitative study

**DOI:** 10.1016/j.socscimed.2020.113034

**Published:** 2020-07

**Authors:** Bethlehem Tekola, Mersha Kinfe, Fikirte Girma, Charlotte Hanlon, Rosa A. Hoekstra

**Affiliations:** a Department of Psychology, Institute of Psychiatry, Psychology and Neuroscience, King's College London, UK; b Department of Psychiatry, WHO Collaborating Centre for Mental Health Research and Capacity-Building, School of Medicine, College of Health Sciences, Addis Ababa University, Ethiopia; c Centre for Innovative Drug Development and Therapeutic Trials for Africa (CDT-Africa), College of Health Sciences, Addis Ababa University, Ethiopia; d Centre for Global Mental Health/King's Global Health Institute, Department of Health Services and Population Research, Institute of Psychiatry, Psychology and Neuroscience, King's College London, UK

**Keywords:** Stigma, Developmental disorders, Parents, Children, Qualitative, Ethiopia

## Abstract

Although stigma related to developmental disorders (DD) has been associated with poor mental health among caregivers, an in-depth understanding of factors that influence internalisation of stigma by caregivers is missing. The aim of our study was to explore perceptions and experiences of stigma among parents of children with DD in Ethiopia and examine the contributing and protective factors for internalised stigma based on the perspectives of the parents themselves. We conducted in-depth interviews with eighteen parents (fourteen mothers, four fathers) in Addis Ababa (between December 25, 2017 and January 8, 2018) and the rural town of Butajira (between August 08, 2018 and August 16, 2018). We analysed the data using thematic analysis. Parents perceived and experienced different forms of stigma that were directed towards their child (public stigma) and themselves (courtesy stigma). Some parents also described how they isolated themselves and their child from social life (affiliate stigma). Parents perceived the negative consequence of stigma on the lives of their child with DD, siblings and themselves. Most parents also described examples of positive reactions and support from their own family and the community. Participants’ accounts suggested supportive contributions and positive responses from the general public came primarily from those who had better awareness of DD. Not all parents in our study internalised the stigma that was directed at them. Whilst perceived family support and acceptance and increased awareness about DD appeared to help some parents not to internalise stigma, the perceived lack of social support and acceptance made some parents vulnerable to internalised stigma. These findings can inform anti-stigma intervention priorities. Awareness-raising activities targeting the community as a whole as well as interventions targeting parents themselves are likely to be beneficial. Interventions should consider the wellbeing of the whole family unit rather than focus on individuals alone.

## Highlights

• Parents of children with developmental disorders in Ethiopia report severe stigma.
• Not all parents in this qualitative study internalised the stigma directed at them.
• Parents also discussed positive reactions and support from others.
• Perceived family support and acceptance helped parents not to internalise stigma.
• Perceived lack of social support made parents vulnerable to internalised stigma.

## 1 Introduction

Stigma has been defined as an “attribute that is deeply discrediting” and that reduces the stigmatised person “from a whole and usual person to a tainted, discounted one” (Goffman, 1963: 3). According to Link and Phelan (2001), stigma exists when labelling, stereotyping, separating, status loss and discrimination occur together in a social, economic and political power situation that allows them. In relation to adult mental health conditions, stigma has also been conceptualised as incorporating problems of knowledge (ignorance), problems of attitudes (prejudice), and problems of behaviour (discrimination) (Thornicroft et al., 2007). Developmental disorders (DD) describe a group of conditions, including autism and intellectual disability (ID), with onset in infancy or childhood that are characterised by impairment or delay in physical, learning, language, or behaviour areas (World Health Organisation, 2013).

Several studies have examined the stigma experiences of caregivers of children with DD across different regions of the world, including East Asia (e.g. Wong et al., 2016), the middle East (e.g. Manor-Binyamini and Shoshana, 2018) and Western contexts (e.g. Broady et al., 2017) (for a comprehensive review, see Papadopoulos et al., 2019 and Liao et al., 2019). Some of these studies were qualitative (e.g. Gray, 2002); many were cross-sectional quantitative studies (e.g. Wong et al., 2016). Findings from the qualitative studies have shown that in addition to the challenges of raising a child with DD (for example, in terms of its impact on caregivers' time, finance and relationships), caregivers experienced stigmatisation (such as rejection, negative judgments and lack of support) within their own family as well as from the wider public (Gray, 2002; Broady et al., 2017). For caregivers of a child with autism, the extent and form of stigmatisation varied depending on the severity of the disorder (Gray, 2002).

A gradually increasing number of studies in Africa (e.g. Aldersey, 2012; Scior et al., 2015; Tekola et al., 2016; Masulani-Mwale et al., 2016; Oti-Boadi, 2017; Gobrial, 2018) have discussed stigma as one of the many challenges experienced by caregivers of children with DD. Manifestations of stigma included negative comments and stares from the wider public (Oti-Boadi, 2017), the use of stigmatising language such as ‘worthless’ and ‘an idiot’ (Tekola et al., 2016), caregivers' (especially mothers') experience of being blamed for their child's DD (Tekola et al., 2016) and discussion of abandonment (e.g. parents abandoning their child because of her/his disorder, or the father abandoning his wife because of the birth of a child with DD) (Aldersey, 2012; Masulani-Mwale et al., 2016). Autism and ID are often attributed to supernatural causes such as curses, possession by evil spirits and punishment from god for the sin that the child's family may have committed (Oti-Boadi, 2017; Gona et al., 2015). Some studies reported that children with ID were killed by those practicing witchcraft (Aldersey, 2012), or caregivers were coerced by neighbours to kill their child (Masulani-Mwale et al., 2016). The few studies that explored the lived experiences of caregivers of children with autism in Africa also highlighted stigma in relation to the social isolation of caregivers of children with autism (Gobrial, 2018; Cloete and Obaigwa, 2019).

In Ethiopia, several studies have examined the stigma related to adult mental health conditions (e.g. Girma et al., 2013), but research exploring stigma related to DD remains scant. Generally, as is the case in other sub-Saharan African countries, there is a paucity of published literature on DD in Ethiopia. The exact prevalence of autism (Elsabbagh et al., 2012) and ID (Njenga, 2009) is unknown in Africa, though is likely to be at least as high if not higher than high-income countries (Maulik et al., 2011). The few published studies on DD conducted in Ethiopia (e.g. Tilahun et al., 2016; Tekola et al., 2016; Zeleke et al., 2018) highlighted lack of service provision for children with DD, lack of awareness and high level of stigma.

Stigma related to DD, particularly internalised stigma, has been associated with poor mental health such as anxiety and depression among caregivers (Papadopoulos et al., 2019; Mitter et al., 2018). But not all caregivers who were exposed to stigmatising attitudes, internalised the stigma and developed poor mental health. In several cross-sectional studies (e.g. Wong et al., 2016), psychosocial mediating and moderating variables such as self-compassion, social support, and caregiver gender affected the process of internalisation of stigma by caregivers. But the quantitative methodology used in these studies precluded an in-depth exploration of how these factors influence the process of stigma internalisation by caregivers.

The aim of our study was to explore perceptions and experiences of stigma among parents of children with DD in urban and rural Ethiopia using a qualitative research approach. In doing so, our study contributes to the existing global stigma literature related to DD by presenting an in-depth account of contributing and protective factors for internalised stigma based on the perspectives of the parents themselves. Our study also presents an in-depth account of what the concept of stigma encompasses in the Ethiopian socio-cultural setting. There is a paucity of research on stigma and stigma reduction interventions in relation to mental health and developmental disorders in low- and middle-income contexts (Thornicroft et al., 2016; Scior et al., 2015) as well as in conditions affecting children and adolescents (Hartog et al., 2019). Thus, our paper contributes to knowledge on stigma in low-resource settings in general and in Africa in particular. This is important because as Kleinman and Hall-Clifford (2009) pointed out any efforts to combat stigma should begin by understanding the social and cultural processes that create stigma in local contexts.

## 2 Methods

### 2.1 Setting

The study was conducted in Addis Ababa (Ethiopia's capital city) and the rural town of Butajira located in the Gurage Zone of the Southern Nations, Nationalities and Peoples' Region (SNNPR). Nearly half of Addis Ababa's population are of the ethnic group Amhara and predominantly Orthodox Christian; Amharic is the dominant language. In Butajira the major ethnic group is Gurage and the population are predominantly Muslim. The major language is Guragigna, but Amharic is also widely spoken. Farming is the main livelihood for people in Butajira while livelihood's in Addis Ababa are more diverse (e.g. daily labour, civil service and industry) (Berhane and Byass, 2002; for more on community variation in SNNPR and Addis Ababa in the context of children and their families, see Pankhurst and Tiumelissan, 2012).

### 2.2 Participants

We approached nine participants in Addis Ababa via a mental health clinic or schools for children with DD and another nine parents in Butajira via a nurse-level health worker. In total eighteen parents (fourteen mothers, four fathers; all parents of different children) participated in our study; all parents who were approached consented to participate. Most of the children (n = 14) had a formal clinical diagnosis of ID. Six children had autism as primary diagnosis; two of these children had a co-morbid formal diagnosis of ID, but the other four children with autism also had some degree of general developmental delay (in Ethiopia autism tends to only be identified if children have more general developmental delays too). Further participant details are presented in Table 1.

**Table 1:** Health and demographic information of caregivers and children who participated in our study.

Caregivers	Main diagnosis of child	Child's age (years) and gender	Caregiver's age (years) and parental role	Caregiver's educational level
C1-A	Autism + ID	4; boy	41; Father	12th grade^a^
C2-A	ID	9; boy	35; Mother	5th grade
C3-A	ID	7; boy	37; Mother	2nd grade
C4-A	Autism	7; boy	40; Mother	No formal education
C5-A	Autism + ID	9; boy	43; Mother	No formal education
C6-A	ID	8; girl	43; Father	12th grade + 3 years further education
C7-A	ID	6; girl	39; Mother	Basic literacy
C8-A	Autism	5; boy	30; Mother	12th grade
C9-A	ID	7; girl	42; Mother	11th grade
C1–B	ID and cerebral palsy	4; boy	29; Mother	8th grade
C2–B	ID; ADHD	9; boy	25; Mother	7th grade
C3–B	Autism	7; boy	30; Father	6th grade
C4–B	ID	7; girl	35; Mother	Degree
C5–B	ID	8; girl	27; Mother	No formal education
C6–B	ID and Epilepsy/Seizure	8; boy	32; Mother	6th grade
C7–B	Autism	9; boy	35; Mother	8th grade
C8–B	ID	8; boy	30; Mother	Basic literacy
C9–B	ID	9; boy	50; Father	8th grade

a completion of 12th grade is equivalent to completion of high school; ID = intellectual disability; ADHD= attention deficit hyperactivity disorder.

### 2.3 Data collection procedures

As part of an ongoing study we interviewed nine parents (seven mothers and two fathers) of children with DD in Addis Ababa between December 25, 2017 and January 8, 2018. We interviewed all parents after they completed participation in a pre-test of the Ethiopian adaptation of the World Health Organization's Caregiver Skills Training (CST) programme (Tekola et al., 2020). The WHO CST teaches caregivers strategies to support their children's social communication and adaptive behaviour and to reduce their challenging behaviour (Salomone et al., 2019). Its content is based on principles of social learning theory, positive parenting, applied behaviour analysis and developmental theories. The programme consists of nine group sessions and three individual home visits, delivered by non-specialists.

The aims of the interviews we conducted in Addis Ababa were to explore parents’ experience participating in the CST and the feasibility and acceptability of the training programme within the local context (see the topic guides Appendix 2). The interviews were not specifically aimed at exploring stigma, but stigma was a prominent theme that came out of our analysis. Eight out of the nine parents interviewed talked about their stigma experiences in connection with questions such as benefits and impact of the programme. With the objective of exploring more about perceptions and experiences of stigma among parents of children with DD, we conducted additional interviews with nine parents (seven mothers and two fathers) from Butajira between August 8, 2018 and August 16, 2018. All interviewed parents in Butajira were also enrolled in a pre-test of the CST programme and had completed six out of the nine CST group sessions at the time of interview.

In-depth-interviews in the local language Amharic were conducted using an interview guide that comprised questions concerning how the parents came to know about their child's DD, parents' help-seeking experiences, experiences of raising a child with DD and their social life (see Appendix 2). In order not to restrict the parents' account of their experiences, we did not use the word stigma in our guiding questions. The first author, who is bilingual in Amharic and English, interviewed almost all parents (seventeen parents). The remaining parent was interviewed by the second author. Both interviewers were independent from the team delivering the CST. While the first author was entirely independent of CST delivery, the second author worked as research coordinator, and therefore had established good rapport with the families prior to the interviews taking place. Before and during our interviews, we attempted to minimise potential power imbalance (e.g. due to our professional background) between researchers and participants. Interviews were conducted at locations preferred by the participants. Prior to the interviews, the interviewers spent time chatting with participants to make them comfortable with the interview process and to build rapport. Participants were treated sensitively, with empathy and care.

### 2.4 Analysis

All interviews were transcribed verbatim in Amharic and translated into English by researchers from Addis Ababa University who are bilingual in Amharic and English. The first author checked the accuracy of all transcripts by comparing them with audio-recorded interviews. Analysis was conducted both in Amharic and English. The first author who led the analysis listened to the Amharic audio-recorded interviews while reading the interview transcripts and noting down initial analytic ideas. All coding in NVivo 11 (QSR International Pty Ltd, 2015) was done on the English translations of the transcripts.

Data were analysed using thematic analysis, using a combination of inductive (‘bottom-up’) and deductive (‘top down’) approaches (Braun and Clarke, 2006:12). The data collected in Addis Ababa were analysed in an inductive way. Specifically, our analysis process involved three main stages. During the first stage, the first author read and re-read transcripts and generated preliminary codes using NVivo 11. During the second stage, the first author met with the last author who independently generated initial codes for three interview transcripts to gain her input. In the third stage, the first author organised all codes into initial themes and sub-themes. Then, the first and last authors met again to review and refine the initial themes and sub-themes by assessing the data associated with each theme. Stigma was one of the six themes that we developed in this inductive thematic analysis process (see Tekola et al., 2020).

When we analysed the interviews that we conducted in Butajira we were already engaged with the stigma literature and our coding and theme development process were guided by existing theories and conceptual frameworks related to stigma. Thus, we used a deductive thematic analysis approach. Specifically, our analysis was informed by Mukolo et al.‘s (2010) framework and the four types of stigma identified in Mitter et al. (2018). Mukolo et al. (2010) presented a framework for conceptualising stigma experience associated with children's mental disorders, incorporating three constructs: a) **dimensions of stigma** (e.g. stereotypes, discrimination, and devaluation), b) **context of stigma** (e.g. self, general public and institutional) and c) **targets of stigma** (e.g. child, family associates, and services). In a systematic review, Mitter et al. (2018) identified four types of stigma relevant to DD. First, **public stigma**, referring to attitudes held within society about stigmatised people. Second, **self-stigma** which is when stigmatised people become aware of and internalise public stigma and consequently withdraw socially and isolate themselves. Third, **family stigma** (also called **courtesy stigma or associative stigma**) indicating stigma experienced by persons associated with the stigmatised person (e.g. their relatives). Fourth, **affiliate stigma** which is when persons associated with the stigmatised person internalise courtesy stigma, resulting in social withdrawal or concealment.

Similar to the stages of data analysis we followed for the data we collected in Addis Ababa, but this time guided by the above discussed broad framework and conceptualisation related to stigma, the first author generated initial codes across all collected data from Butajira, using NVivo 11. The first author then shared the initial codes and associated data extracts with the last and second authors. The last and second authors then reviewed all the codes and data extracts against all interview transcripts and provided their input. The first author modified and expanded initial codes based on the co-authors’ comments.

Finally, the first author developed themes and sub-themes from coded data related to stigma (Addis Ababa) and all coded data (Butajira). These initial themes were revised and refined several times by the first author in relation to the coded and collected data following further input from the second, fourth and last authors, and in light of the above discussed broad framework and conceptualisation related to stigma before the final four themes and sub-themes were produced.

### 2.5 Ethical considerations

The study received ethical approval from the institutional review board of the College of Health Sciences of Addis Ababa University (reference 062/16/Psy) and from the Psychiatry, Nursing and Midwifery subcommittee of King's College London's College Research Ethics Committee (reference HR-16/17–3489). All participants provided informed consent.

## 3 Findings

Our thematic analysis resulted in the generation of four themes and sub-themes:

1. Dimensions of parents' perceived stigma experiences
  ⁃ Public stigma
  ⁃ Courtesy stigma
  ⁃ Affiliate stigma
2. Perceived consequence of stigma
3. Parents' positive social experiences/or lack of stigma
4. Factors influencing affiliate stigma
  ⁃ Perceived family support and acceptance
  ⁃ Increased awareness about DD

Below we present each of the themes and sub-themes with selected data extracts to support our interpretations. The remainder of the data extracts including full quotes from all participants relating to each theme and subtheme can be found in the supplementary materials (Appendix A). Quotes from participants from Addis Ababa are denoted with ‘U’ to mean urban; quotes from parents from Butajira are presented with ‘R’ to mean rural.

### 3.1 Dimensions of parents’ perceived stigma experiences

This theme captures the different forms of stigma experiences reported by parents during the interviews. The theme is organised around three sub-themes, representing three of the four core forms of stigma often mentioned in DD stigma literature.

#### 3.1.1 Public stigma

This sub-theme covers parental accounts of the negative attitudes and responses that their child with DD experienced within the wider community. Parents talked about the negative stereotypes that the public held about their child with DD. In the quote below, a mother of a seven-year-old girl with ID explained how people see her daughter as dangerous and a threat to their child's safety, even if her daughter behaved and acted to the contrary:

…I am living in a community which does not understand you [children with DD and their caregivers]. My daughter, for example, fears fight, she is calm and doesn't touch anyone and she also doesn't want to be touched by others, but the community sees her in another way. They think she will hit and push their children. There is exclusion … (Caregiver 9-U)

Parents suggested fear was an important factor explaining why other children avoid their child with DD, as illustrated in the following quote from a mother of a four-year-old boy with ID and cerebral palsy:

…kids don't know anything, when they see him, they run. He also runs with them thinking that it is part of the play, but they run because they fear him … (Caregiver 1-R)

Children with DD also experienced stigma in the form of not being accepted into other children's games, as a father of a seven-year-old boy with autism explained:

I try to make him play with kids, but when I see that they talk and play together but he can't do that, and I sometimes feel bad when they neglect him. When I feel that way, I take him out and try to have him play with leaves … (Caregiver 3-R)

Some parents indicated that they encountered staring and pity from the public when they are out with their child, as the mother of a seven-year-old girl with ID explained:

There is a problem. I don't like it when people pity her. I say to them: ‘what is wrong with her?’ and I even try to fight with them. On the taxi and on the street when people stare at her I say: ‘what are you looking at?’ May God give them understanding … (Caregiver 4-R)

#### 3.1.2 Courtesy stigma

In addition to stigmatising experiences directed towards their child with DD, parents also talked about how they themselves were socially excluded because of their child's DD. In the quotes below, two mothers explained how their family's perceived embarrassment of being associated with a child with DD and fear of losing dignity in the community has effectively resulted in their exclusion:

P.…I am excluded by my family including my mother.

I. What did they say?

P. Their problem is embarrassment. They told me not to bring my child to their house during daytime [when people can see her] and they told me to bring her to their house during the night-time [so that nobody can see her]. But only Satan moves at dark. We, children of God, will move in the daytime. They are worried about their dignity. (Caregiver 9-U).

…my mother, I have told you, she used to say take him [my child] to the back of the house when someone comes to the house. And my biggest fight with my mother was: ‘why do you say that; it is not good’. They only think about the family [name] …. (Caregiver 1-R)

Parents also stated that they experienced negative comments and teasing from their neighbours and were blamed for their child's DD, as a mother of a nine-year-old boy with ID commented:

The attitude of other people, because x [my child] can listen but can't talk, they say, why doesn't your child talk. My neighbours used to say my child is *Duda* [tongue-tied]. They were saying, did you give birth to a *Duda*? They were teasing me. They were making fun of me saying her oldest child is like this. They were saying I must have done something [to cause the child's condition]. They were saying all of this in my presence, but I used to pretend that I was not listening but cry when I got home … (Caregiver 2-R)

Some parents explained how their own family and the public considered their child's DD as a curse, resulting in stigmatising attitudes as a mother of a four-year-old boy with ID explained:

…once in a taxi, I was holding him [my child] when I get into the taxi. It was an old lady, she looked shocked and said, ‘in the name of the father.’ I asked her what was wrong because I was shocked [by her reaction] too. She said that this thing [my child's condition] was a curse and that I should ask my family. I cried a lot until I couldn't talk … (Caregiver 1-R)

A mother of a nine-year-old boy with autism expressed a lack of support and rejection she experienced from her husband because of his lack of acceptance of their child's condition:

…because my child is like this my husband was even asking me to leave. We were about to divorce. He was even saying: ‘I don't want to see your eyes take your child and leave’. I was not telling this to my family. I was just crying … (Caregiver 7-R)

#### 3.1.3 Affiliate stigma

Some parents talked about how they isolated themselves and their child with DD from social life. For some, this was because they found it difficult to manage their child in social situations, as a mother of a seven-year-old boy with autism commented:

I don't meet up with neighbours. I don't mix up with anybody. I sit at home with the child. When I go with the child to places, he does not sit down. He disturbs me. I don't mix up with people. I don't meet my relatives anymore. I don't mix up with anybody. I don't mix up with neighbours. I don't mix up with anybody. When my [other] children come [from school] I open the door for them and then close the door. (Caregiver 4-U)

For others it was to protect themselves from the further negative consequence of public stigma on their mental health, as a mother of a seven-year-old girl with ID explained:

The awareness level of the community is very low, and a lot needs to be done. They don't share their thought with you. They talk about you behind your back and because of that, you will be forced to exclude yourself from them. That is because … we are not living with educated people. They believe in the curse and they give different explanations. Due to this and to protect your mind you will exclude yourself. It has a huge impact. It is very difficult. (Caregiver 9-U)

Fearing the prospect of stigma based on previous stigmatising experience also made some parents isolate themselves. The mother of a nine-year-old boy with ID who stated that her neighbours teased her for having a child who does not speak noted: “If I want to take him [my child] to social places this thing [the negative reaction of her neighbours], this feeling will come to my mind” (Caregiver 2R).

Table 2 in the supplementary material presents the different dimensions of stigma as reported by caregivers in this study, as well as the context and targets of the stigma experience.

### 3.2 Perceived consequence of stigma

Parents perceived the negative consequences of stigmatising experiences on the lives of their child with DD, their siblings and themselves. Some parents were particularly worried about the effect of negative attitudes and responses towards their child on the child's mental health, as a mother of a four-year-old boy with ID explained:

…the thing that I always ask myself is what is the effect of the community's attitude on the child later. I swear to Allah I am very worried. I am worried that this may lead him to become mad … (Caregiver 1-R)

Another mother of a child with ID noted that the stigmatising experiences towards her child with DD have made her worried about who is going to look after him after she died: “…nobody will look after him. People are avoiding him even when I am here. If I die nobody will care about him …” (Caregiver 2-R). Some parents also talked about how stigma affected siblings in the form of showing negative emotional responses (“gets uncomfortable”, “gets upset”) as the following quote from a mother illustrates:

…if we are three, me, my older one and my youngest, the older one gets uncomfortable because they [people] stare at his youngest brother, he asks me why they stare, and he gets upset. I tell him to ignore them … But I feel it inside me, I can imagine how he is feeling … (caregiver 1-R)

In addition to experiencing negative emotional reactions as a result of stigma, parents also explicitly talked about how the stigma they experienced affected them as two mothers of children with ID indicated: “this still hurts me” (Caregiver 2-R), “I cried until I couldn't talk” (Caregiver 1-R). Parents described how the stigma they experienced prevented them from taking their child out and forced them to isolate themselves from the outside world:

…truly, people are killing what is left of my energy. They are just making me be ashamed when I decide to take him [my child] out … I am starting to believe that in rural areas the mothers who hide their children behind closed doors are right. That is because this [mothers hiding their children] is a result of the public's responses. Tomorrow, when I take my child out what could happen, will be worse … (Caregiver 1-R)

### 3.3 Parents’ positive social experiences/or lack of stigma

Despite parents’ perceptions and experiences of stigma towards their child and themselves, the accounts of many parents also suggested that they received support and understanding, not only from their own family, but also from their neighbours, schoolteachers and the general public. In the quote below, a mother of an eight-year-old boy with ID explained how she was positively treated:

I have not experienced stigma so far. People help me. I have not experienced such a thing … [for example] when I take my child to Black Lion Hospital people let me get into the taxi without queuing when they see my child biting me (Caregiver 6-R)

Parents emphasised that, among the general public, those who had positive reactions and understood their situation were people who had better awareness of DD. A mother of a nine-year-old boy with ID explains:

We got into a bus once and he [my child] snatched a hat from an older person, this person did not know about my son's condition. When my son took his hat, that person took the hat back and hit him. Another person, I think he knows about such children, asked that older person if it was appropriate to do that [hit the child] and they got into an altercation …. (Caregiver 2-U)

Based on her previous stigma experience in public places a mother of a nine-year-old boy with ID reflected:

If people know they would not say to me such [negative] things. People who know say ‘leave him alone’. People who have awareness understand when he exhibits difficult behaviour on the street … but most people do not understand and that is why I don't take him to social places (Caregiver 2-R)

Another mother noted that when her child behaved in a socially inappropriate manner on public transport, she received support from a passenger whom she explained her child's condition to, suggesting that lack of knowledge about the problem contributed to people's negative attitudes and responses:

I: How is the attitude of people in other places?

P. One day when we go to Yekatit Hospital when he [my child with DD] was wrestling, one person warned me not to go with my child on a taxi next time.

I. Who was that person?

P. He was the driver assistant and the person sitting next to me asked me [about my child] and I told him that he has a problem. Then, he was about to fight with the assistant saying that he has a problem, he has autism … (Caregiver 3-U)

Some parents also talked about the support and understanding they received from their family and neighbours. A mother of a seven-year-old girl with ID said:

Thank God, she [daughter with DD] has increased love among us [me and my husband]. What is seen in some families is rift [between husband and wife]. But here the love she has for her father is special. He is also good. Thank God. God has not disappointed me in this regard. While he gave me a child like her, he gave me a good husband … my family supports me. My mother lives a bit far from here, but she supports me morally. When I take my child to her place, I feel uncomfortable thinking that people may see her. My mother and sisters are not like that. They take her out … (Caregiver 4-R)

### 3.4 Factors influencing affiliate stigma

The accounts of parents in both sites suggest that not all parents who perceived and experienced stigmatising attitudes went on to internalise the stigma. Under this theme, we discuss a contributing factor for internalised stigma among parents and two factors that seemed to help parents not to internalise stigma.

#### 3.4.1 Perceived family support and acceptance

The parents' reports suggest that perceived availability or lack of family support and acceptance made a difference to whether parents internalised stigma and withdrew socially or not. Those parents who said that their family supported and accepted them indicated they did not feel embarrassed or ashamed despite experiencing stigma and emphasised they did not restrict their activities with their child nor avoided going to public places. In the first quote below, a mother of a nine-year-old boy with autism and ID explained how her family accepted her and her child's condition. Elsewhere in her interview (in the second quote) she also discussed how the stigma she experienced from the public did not stop her from doing whatever she wanted to do with her child or changed her feelings:

My family members have a good understanding of my child's problem. Due to this they are giving him good love and care more than me. So, my family believe this and accepted me well.

As you know the thing about our community is, they talk behind your back when you go out with the child or when the child does something. But that does not stop me. I don't feel ashamed. I go with him to the shop. I go with him to the church. I take him everywhere I want. People can say different things, but I don't give much attention to that. (Caregiver 5-U)

Another mother of a seven-year-old girl with ID talked about how initially the stigma that she experienced made her to feel embarrassed and led to self-blame, but how her family's emotional support helped her to stop this negative self-evaluation:

It is very difficult. You would take your child to the church and you would feel embarrassed. I used to think as if I am different. I used to say: ‘what have I done wrong?‘. In this regard, I want to thank my family and my husband very much. They say: ‘you have not done anything wrong. you have been told; you know it’ … (Caregiver 4-R)

On the other hand, those parents who said that they did not get support and acceptance from their family reported feelings of shame and self-blame and withdrew themselves and their children socially. In the quote below, a mother of a seven-year-old girl with ID talked about how she did not get acceptance from her family and how she was forced to accept the stigmatising experiences she and her daughter were facing and isolated herself:

There is a huge pressure. There is exclusion starting from my family and there are very difficult situations, but I don't have a choice but to accept that. There is a problem when we go on the road and when people see us and even in our family. You will be excluded eh … especially from my family, nobody accepted me including my mother. Because of this, I am not living in the community. I am living only with my child … (Caregiver 9-U)

Another mother of a nine-year-old boy with autism talked about how she did not get support from anybody including her husband, how she blamed herself and isolated herself socially until recently, fearing the prospect of people's negative responses:

I don't have anybody who helps me except God. It is me who does everything even my husband does not support me [crying] …. I feel very sorry for him [my child] when I see him being inferior to his friends [other children]. I say: what have I done wrong to God? [crying] … up until he was six-year-old, he peed and pood on himself. I did not mix with people thinking that they may be disgusted. I felt like all people walking on the street are only looking at me …. (Caregiver 7-R)

#### 3.4.2 Increased awareness about DD

Some parents' accounts in both sites suggest that their increased awareness about their child's DD changed how they perceived other people's negative attitudes and responses. Below a mother explained how increased knowledge about DD helped her to disclose her child's condition to other people and gained confidence:

When I first knew about my child's condition, I used to not talk about it. When neighbours asked me about my child, I used to say she has a heart problem and did not say she has a developmental problem. I considered it as something bad happened to me. I did not talk about it. It is only recently that I started to talk about it … after I became aware, I started to talk with confidence. (Caregiver 4-R)

Another mother also explained how the CST training she took changed her reaction to her neighbours' and other people's negative attitudes suggesting that the training may have helped in terms of changing her perception:

I: Tell me about the benefit of this training for you. For example, what changes have come because you took the training?

P. My neighbours may say many things, but I didn't get upset and cry like the previous times because I took the training.

I. What did they say, for example?

P. as you know, people say many things. They consider it as a curse, and they say many other things and give many suggestions. Due to this, I cried many times before I took this training but now, I didn't cry after I took this training. I didn't say anything when they say different things and I prefer to be silent. This is the result of the training. (Caregiver 7-U)

## 4 Discussion

### 4.1 Overview of findings

The aim of this paper was to explore how parents of children with DD in urban and rural Ethiopia perceived and experienced stigma. To help us with this exploration, we used Mukolo et al.‘s (2010) framework and the four types of stigma identified in Mitter et al. (2018). Our findings lend support to Mitter et al. (2018) and Ali et al. (2012) who found that caregivers of individuals with DD perceived and experienced courtesy stigma both within their families and communities, although the extent and form of stigma experienced varied across cultures (see also Scior, 2016; Liao et al., 2019). Our study went beyond reporting courtesy stigma by highlighting stigma experiences directed at the child with DD (public stigma) as perceived by their parents. Some parents in our study also described how they isolated themselves and their child with DD from social life (affiliate stigma) (see also Dababnah and Parish, 2013; Broady et al., 2017).

The lived experiences and perceptions of parents in this study also provided an in-depth understanding of what constitutes stigma in this socio-cultural setting. Specifically, Kleinman and Hall-Clifford's (2009: 3) notions of ‘the unique social and cultural processes that create stigma’ and the contexts within which people stigmatise and are stigmatised (Yang et al., 2007). Some mothers in our study, both rural and urban, talked about being excluded by their family members (specifically by their mother) because of the embarrassment of being associated with a child with DD, saying their family were “worried about their dignity” or “they only think about the family [name]”. In line with Yang and colleagues' (2007: 1525) conceptualisation of stigma as a ‘moral experience’ in which stigmatised conditions threaten ‘what is most at stake for actors in a local social world’, we argue that what was threatened in our participants' and their family's local world was **family honour and pride.**

One of the very few ethnographic studies relating to Ethiopia, Heinonen (2011: 32–36) found that the ‘wider Ethiopian culture’ is ‘shame-based’. She noted despite ethnic and religious diversity, *Yilunta* which is associated with **shame, honour and family pride** is one of the culture protocols which define Ethiopian culture. According to Heinonen, *Yilunta* which incorporates ‘sensitivity to and concern about the opinion of others’ (‘what would others say’) and ‘a deeply rooted concern with status and family pride’ is central to Ethiopians' identity. Ethiopian children are socialised to internalise a deep sense of *Yilunta* from young age (e.g. they are taught to strive not to shame their family). Central to conforming to *Yilunat* is the need for the children to learn to live in harmony with other people such as neighbours. She found in practice what *Yilunta* involves differs based on age, gender and socio-economic status. And like other cultural codes *Yilunta* is subject to change, modification and continuity. In our study, by excluding their daughter who has a child with DD the mothers were avoiding shame and protecting the family's honour and pride which is vital for maintaining relationship with the community around them. Some parents' account of not wanting to be pitied is also related to the importance of being proud, showing pride and not showing that one is ‘in need of anything’ in the Ethiopian culture (Molvaer, 1995:103). This is exemplified in the Amharic proverb “pride is worth a dinner” which means that one's image or standing in society is more valuable than immediate self-gratification. The metaphor of supper refers to temporary benefit, something that may bring pleasure or temporary satisfaction. Thus, what is advised is self-restraint, even if that self-restraint would put one in an uncomfortable situation temporarily. Our data suggests that in relation to DD mothers were more likely to be shamed and are also more likely to have feelings of shame than fathers. This may be related to the fact that Ethiopian mothers are seen as major gatekeepers of local norms and practices (Poluha, 2004) or that it is primarily seen as a mother's responsibility to raise and socialise children.

Disability including ID is linked with shame in Ethiopia (Teferra, 2005). Parents are often hesitant to involve their child with disability in the immediate community as they are seen to bring shame upon the family (Schiemer, 2017). Traditionally, disability (Teferra, 2005) and mental illness (Patel, 1995) are attributed to supernatural phenomena such as curses, spells, being possessed by evil spirits and punishment by Supreme Being (e.g. God, Allah, Waqa) for sins. Compared to other physical and mental health conditions, ID is particularly more likely to be seen as having spiritual and magical origin (Mulatu, 1999; Tilahun et al., 2016). Persons with mental health conditions and their families are seen as carrying some responsibility for the health condition e.g. by failing to fulfil religious duties (Monteiro and Balogun, 2014). In our study, attributing DD to curses was prominent in a number of participants' accounts and some mothers said they were blamed for their child's condition.

Several quantitative studies indicate that there is an association between stigma and caregivers' mental health, including depression, anxiety and psychological distress (see Papadopoulos et al., 2019). Stigma has also been found to play a major role in caregivers’ overall life burden (Kinnear et al., 2016). In our study, parents perceived the negative consequence of stigma on the lives of their child with DD, siblings and themselves. A number of qualitative studies (e.g. Dababnah and Parish, 2013) and few cross-sectional studies (e.g. Tilahun et al., 2016) also reported the effects of courtesy stigma on negative emotions and social isolation. However, the consequence of stigma on siblings is hardly studied (Mitter et al., 2018).

In addition to detailed accounts of stigma experience, most parents in our study described examples of positive reactions and support from others, including from family members, neighbours, schoolteachers and the general public (see also Dababnah and Parish, 2013). Parents in our study emphasised that, among the general public, those who had positive reactions and understood their situation were people who had better awareness of DD. They explained that people who have awareness were tolerant and did not react negatively when their child acted in a socially inappropriate manner in public places.

Not all parents in our study internalised the stigma that was directed at them. The parents' accounts provided clues as to which factors may contribute or protect against internalised stigma (affiliate stigma). Whilst perceived family support and acceptance and increased awareness about DD appeared to help some parents not to internalise stigma, the perceived lack of social support and acceptance made some parents vulnerable to internalised stigma. Werner and Shulman (2013) similarly found that self-esteem, social support and positive meaning in caregiving moderated the association between affiliate stigma and subjective wellbeing in caregivers. Mitter et al. (2018) also emphasised the role of a supportive network and acceptance in alleviating the adverse effects of stigma in family members of people with DD. But given that most of these studies are cross-sectional in design, they are unable to tell us anything definitive about causal order in the social support/affiliate stigma relationship.

### 4.2 Implications

The findings of this study have a number of implications for research and practice. Theoretically, we found Mukolo et al.‘s (2010) broad framework which incorporates three constructs (dimensions of stigma, context of stigma and targets of stigma) and the four types of stigma identified in Mitter et al. (2018) relevant to conceptualise the stigma experiences of children with DD and their parents in Ethiopia. However, the contents of each of the three constructs and the types of stigma reflected the culture and context in which participants live. Our findings suggest that a broader conceptualisation of stigma that incorporates social, cultural and moral components as proposed by Kleinman and Hall-Clifford (2009) is more suitable for the Ethiopian context.

The accounts of parents suggested that lack of knowledge and understanding about DD contributed to people's negative attitudes and responses towards their child with DD and themselves. Thus, awareness raising activities targeting the community as a whole are likely to be beneficial. Previous work in Ethiopia suggests that a brief training intervention for community health workers decreases negative beliefs and stigmatising attitudes about children with DD (Tilahun et al., 2019). In our study, increased awareness about DD among parents appeared to play a positive role in helping some parents not to internalise stigma. This means awareness-raising activities that target parents themselves (e.g. as included in CST) are also vital. Furthermore, parents' accounts suggested that stigma had adverse effects not only on the lives of their child with DD but also on siblings and themselves. Therefore, interventions should consider the wellbeing of the whole family unit rather than focus on individuals alone.

In discussing factors that influence the relationship between stigma and mental health outcomes among caregivers of children with autism, Papadopoulos et al. (2019), differentiated between protective factors that can be changed through research interventions (e.g. caregiver self-esteem, self-compassion) and those that cannot be changed through interventions (e.g. gender and culture). They highlighted the importance of targeting changeable protective factors (for example, by strengthening support structures) to support and protect caregivers from stigma and enhance their mental health. Previous research (e.g. Evans-Lacko et al., 2012) has also shown that there is a direct connection between public stigma and internalised stigma: when public stigma decreases, internalised stigma also decreases. Therefore, in order to reduce internalised stigma (affiliate stigma) in parents of children with DD, in addition to enhancing protective factors in caregivers, interventions should focus on reducing public stigma and increasing social support. Interventions that consider broader socio-structural factors such as poverty are also needed, as individual and family level interventions for internalised stigma may put the burden of change on the stigmatised and their families (Pantelic et al., 2019).

### 4.3 Strengths and limitations

A strength of this study is that it is the first in Ethiopia and in Africa overall to consider the broad range of parents' stigma experiences related to DD and factors that influence internalisation of stigma by parents foregrounding the perspectives of the parents themselves. A further strength is the inclusion of participants living in a rural town, most of whom had a child who was only recently diagnosed with DD as part of the CST study. As such these families are similar to the majority of families with a child with DD in sub-Saharan Africa, most of whom remain without a formal diagnosis. Their accounts are likely to be more reflective of most African experience than accounts from parents who have known their child's DD diagnosis for a long time and have had time to grapple with what that means.

Parents were interviewed as part of the CST project, i.e. all parents were enrolled in training, something unavailable to most families in Africa. This could be considered a limitation of our study as participation in the CST may have affected caregivers' perspectives on stigma. The study only included four fathers, limiting our ability to explore differences in stigma experiences between fathers and mothers. We also could not make meaningful urban-rural comparison on parents’ stigma experiences because of the imbalance in the depth of data on stigma we collected from Addis Ababa and the rural town of Butajira. Unlike the interviews in Addis Ababa, mainly related to the CST programme (stigma only came out inductively from our analysis), the interviews conducted in Butajira explicitly focused on exploring perceptions and experiences of stigma.

Furthermore, it is important to acknowledge that many of the existing conceptual understanding of stigma place a heavy emphasis on individual beliefs and actions which can overshadow broader political and economic issues (e.g. poverty and war) that shape stigmatising attitudes and beliefs (Tyler and Slater, 2018). We found the concept of stigma useful as a starting point to analyse parents' perceptions and experiences of negative attitudes and practices towards their child with DD and themselves within the family and broader community contexts. But future research is needed that combines parents’ perceptions and experiences of stigma with considerations of broader socio-political and economic factors affecting the lives of their child with DD and themselves.

## 5 Conclusions

Parents of children with DD in Ethiopia perceived and experienced various forms of stigma that were directed towards their child with DD and themselves from their family and the general public. Parents further discussed the negative consequences of stigma on the lives of their child with DD, siblings and themselves. Not all parents in our study internalised the stigma that was directed at them. It appears that perceived family support and acceptance and increased awareness about DD helped some parents not to internalise stigma. Conversely, perceived lack of social support and acceptance made some parents vulnerable to internalised stigma. Parents’ accounts in this study also reflected their positive social experiences.

## CRediT authorship contribution statement

**Bethlehem Tekola:** Conceptualization, Methodology, Investigation, Formal analysis, Writing - original draft. **Mersha Kinfe:** Investigation, Formal analysis, Writing - review & editing, Project administration. **Fikirte Girma:** Conceptualization, Resources, Writing - review & editing. **Charlotte Hanlon:** Conceptualization, Methodology, Formal analysis, Writing - review & editing, Supervision. **Rosa A. Hoekstra:** Conceptualization, Methodology, Formal analysis, Writing - review & editing, Supervision, Funding acquisition.

## Footnotes

Appendix A Supplementary data to this article can be found online at https://doi.org/10.1016/j.socscimed.2020.113034.
