# Supplementary material for: Perceptions and experiences of stigma among parents of children with developmental disorders in Ethiopia: A qualitative study
Source: Soc Sci Med. 2020 Jul;256:113034. doi: 10.1016/j.socscimed.2020.113034 (PMC7322556; doi:10.1016/j.socscimed.2020.113034)
Supplement: Multimedia component 1 [file mmc1.docx]

**Appendix A: Supplementary data**

Table 2: Dimensions of stigma

| **Dimensions of stigma** | **Example quotes** | **Context of stigma** | **Targets of stigma** |
| --- | --- | --- | --- |
| Avoiding | People are avoiding him [my child] even when I am here [alive]. If I die nobody will care about him… (Mother of a nine years old boy with ID and ADHD, rural) | Public | Child |
| Avoiding | …kids don’t know anything, when they see him [my child], they run. He also runs with them thinking that they are playing. But they run because they fear him… (Mother of a four years old boy with ID and cerebral palsy, rural) | Public [other children] | Child |
| Exclusion | Last time, x [a protestant church] was registering children to give education. And I was told to bring him [my child] as well. I took him hoping that he will spend time with other children and be able to learn. Even though I am a Muslim I don’t have a problem with the religion… I only wanted my child to spend time with other kids and be able to learn. But once they realised that he can’t speak they refused to register him. I was willing to pay them as much as I can afford but they said no while all his friends got registered. I felt sad that day… (Father of a seven years old boy with autism, rural) | Church [institution] | Child |
| Avoiding | I try to make him play with kids, but when I see that they talk and play together but he can’t do that, and I sometimes feel bad when they neglect him when I feel that way I take him out and try to have him play with leaves… (Father of a seven years old boy with autism, rural) | Public [other children] | Child |
| Avoiding | …the children also fear her. They will run away saying ‘here she comes’… (Mother of an eight years old girl with ID, rural) | Public [other children] | Child |
| Seeing child with DD as dangerous/violent leading to exclusion | …I am living in a community which does not understand you. My daughter, for examples, fears fight, she is calm and didn’t touch anyone and she also didn’t want to be touched by others, but the community saw her in another way. They think she will hit and push their children. There is exclusion… (Mother of a seven years old girl with ID, urban) | Public | Child /parent |
| Stares | …for instance, when I take out [my child], just ask me who doesn’t turn around to look at him, everyone turns around to look at him, that is just it. I just suggest to them to watch where they are going so that they won’t fall. That is what I mean, there is no one who doesn’t turn to look at him… (Mother of a four years old boy with ID and cerebral palsy, rural) | Public | Child/parent |
| Stares  Pity | There is a problem. I don’t like when people pity her. I say to them: ‘what is wrong with her?’ and I even try to fight with them. On the taxi and on the street when people stare at her I say: ‘what are you looking at?’ May God give them understanding… (Mother of a seven years old girl with ID, rural) | Public | Child/parent |
| Rejection  Lack of support and acceptance | …because my child is like this my husband was even asking me to leave. We were about to divorce. He was even saying: ‘I don’t want to see your eyes take your child and leave’. I was not telling this to my family. I was just crying… (Mother of a nine years old boy with autism, rural) | Husband | Parent/child |
| Considering it as a curse | …once in a taxi, I was holding him [my child] when I get into the taxi. It was an old lady, she looked shocked and said in the name of the father. I asked her what was wrong because I was shocked [by her reaction] too. She said that this thing [my child’s condition] was a curse and that I should ask my family. I cried a lot until I couldn’t talk … (Mother of a four years old boy with ID and cerebral palsy, rural) | Public | Parent |
| Considering it as a curse | My family sees it as a curse… this still hurts me… because he is my first child, I tried to ignore what people say. But I say to myself Allah [God] gave me this child, may He be my vindicator and give me justice… (Mother of a nine years old boy with ID and ADHD, rural) | Family | Parent |
| Embarrassment/shame leading to exclusion | …my mother, I have told you, she used to say take him [my child] to the back of the house when someone comes to the house. And my biggest fight with my mother was: ‘why do you say that; it is not good’. They only think about the family [name]…. (Mother of a four years old boy with ID and cerebral palsy, rural) | Family | Child/parent |
| Labelling (seeing it as illness) | When his mother and I take him to his grandmother’s area when they [people] say ‘is he better now’. He has nothing wrong except that he does not speak…his mother and I understand him, what he wants. Other people may not understand him and think that he is ill. When they say ‘is he better now’ that makes me sad. (Father of a seven years old boy with autism, rural) | Public | Parent |

Note: ID= Intellectual disability; ADHD= Attention deficit hyperactivity disorder;

DD= Developmental disorders

**Theme 1: Dimensions of parents’ perceived stigma experiences**

| **Participants** | **Dimensions of parents’ perceived stigma experiences** | | |
| --- | --- | --- | --- |
|  | Public stigma | Courtesy stigma | Affiliate stigma |
| Caregiver 1-A; Father, Urban | …if you see his face, my child is very handsome. He is very handsome. I am not exaggerating but he is handsome, has beautiful eyes and teeth and he has also a good physical structure. But he can’t speak. I know he shows some symptoms. I can’t take him to relatives and neighbours. If you ask me why? It’s because he can’t speak and when he tries to speak, he screams and he says like, ehh...ehh and the neighbours are not aware of such type of behaviour, they don’t have the understanding... | I am not talking with neighbours or other people that my child has this type of case, I don’t want to let them to blame me. When they ask me why my child is not talking, I tell them that it takes time to get an improvement. I tell them that, it’s not possible to have a radical change. That’s how I try to clarify things for them but it’s still challenging… | …I don’t care about what people say or don’t say. But, regarding my child, I need him to grow up freely. I don’t hide him from people just because people might say something about him, I try to make him to mix up with people. Of course, my wife doesn’t want him to get close to people, I don’t want to deny that. That is because she is afraid that people might say something about him and exclude him. But I always want him to be free. Sometimes, we may argue because she doesn’t want him to get out of the house, but I want him to get out of the house because he should experience everything, the sun, the cold weather so that it won’t be a new thing for him. But people still have a problem. |
| Caregiver 2-A; Mother, Urban | On the road, on transport people don't usually understand. There is also a big problem in social life… We got into a bus once and he [my child] snatched a hat from an older person, this person did not know about my son's condition. When my son took his hat, that person took the hat back and hit him. Another person, I think he knows about such children, asked that older person if it was appropriate to do that [hit the child] and they got into an altercation…. And all these has a big influence on social life… | Even in your family a normal child and a sick child are not seen equal because they don't have the awareness. They don't see it as a health condition. They think it is because you [parents] have committed a sin [ and as a result have this kind of child]. In the neighbourhood, in the family, wherever you go, there is a lot of burden. |  |
| Caregiver 3-A; Mother, Urban |  | I: How is the attitude of people in other places?  P. One day when we go to Yekatit Hospital when he [my child with DD] was wrestling, one person warned me not to go with my child on a taxi next time.  I. Who was that person?  P. He was the driver assistant and the person sitting next to me asked me [about my child] and I told him that he has a problem. Then, he was about to fight with the assistant saying that he has a problem, he has autism… Then, after this person got off from the taxi, the assistant was saying to me you were about to make me hit by that person. I tried to ignore him. I have faced such kind of problem. |  |
| Caregiver 4-A; Mother, Urban |  |  | I don’t meet up with neighbours. I don’t mix up with anybody. I sit at home with the child. When I go with the child to places, he does not sit down. He disturbs me. I don’t mix up with people. I don’t meet my relatives anymore. I don’t mix up with anybody. I don’t mix up with neighbours. I don’t mix up with anybody. When my [other] children come [from school] I open the door for them and then close the door. |
| Caregiver 5-A; Mother, Urban | In the previous times, this community excludes people with HIV/AIDS. Similar to that, the community doesn’t want these children [children with developmental disorder] to touch and push their children. I don’t worry about this. He [my son] plays with his siblings at home. |  |  |
| Caregiver 6-A; Father, Urban |  |  |  |
| Caregiver 7-A; Mother, Urban |  |  |  |
| Caregiver 8-A; Mother, Urban |  | There aren't that much people that openly tell you. You don't know. Lots of people don't openly tell you but some people, tell me to take him to the holy water place and the Muslims tell me to take him to the house of Quran. Be that as it may, in line with my religion I take him because anyone even people who are healthy need it. So, I take him. But they tell you that he will be healed that way. Some people talk to you about it but all of them talk behind your back. |  |
| Caregiver 9-A; Mother, Urban | . You told me about your relationship with you family earlier. How is your relationship with your neighbours?  P. It is unthinkable.  I. Unthinkable?  P. Yes, it is unthinkable. You know why it is because I am living in a community which does not understand you. My daughter, for example, fears fight, she is calm and doesn’t touch anyone and she also doesn’t want to be touched by others, but the community sees her in another way. They think she will hit and push their children. There is exclusion. They don’t understand you. In this kind of situation, it is difficult for your mind. | P.…I am excluded by my family including my mother.  I. What did they say?  P. Their problem is embarrassment. They told me not to bring my child to their house during daytime [when people can see her] and they told me to bring her to their house during the night-time [so that nobody can see her]. But only Satan moves at dark. We, children of God, will move in the daytime. They are worried about their dignity. | The awareness level of the community is very low, and a lot needs to be done. They don’t share their thought with you. They talk about you behind your back and because of that, you will be forced to exclude yourself from them. That is because…we are not living with educated people. They believe in the curse and they give different explanations. Due to this and to protect your mind you will exclude yourself. It has a huge impact. It is very difficult. |
| Caregiver 1-B; Mother, Rural | …kids don’t know anything, when they see him, they run. He also runs with them thinking that it is part of the play, but they run because they fear him…  …for instance, when I take out [name child], just, ask me who doesn’t turn around to look at him, everyone turns around to look at him, that is just it, I just suggest to them to watch where they are going so that they won’t fall down, that is what how I mean, there is no one who doesn’t turn to look at him | …my mother, I have told you, she used to say take him [my child] to the back of the house when someone comes to the house. And my biggest fight with my mother was: ‘why do you say that; it is not good’. They only think about the family [name]….  …once in a taxi, I was holding him [my child] when I get into the taxi. It was an old lady, she looked shocked and said, ‘in the name of the father.’ I asked her what was wrong because I was shocked [by her reaction] too. She said that this thing [my child’s condition] was a curse and that I should ask my family. I cried a lot until I couldn’t talk … |  |
| Caregiver 2-B; Mother, Rural |  | The attitude of other people, because x [my child] can listen but can’t talk, they say, why doesn’t your child talk. My neighbours used to say my child is Duda [tongue-tied]. They were saying, did you give birth to a Duda? They were teasing me. They were making fun of me saying her oldest child is like this. They were saying I must have done something [to cause the child’s condition]. They were saying all of this in my presence, but I used to pretend that I was not listening but cry when I got home…  My family sees it as a curse… this still hurts me… because he is my first child, I tried to ignore what people say. But I say to myself Allah gave me this child. May He be my vindicator and give me justice… |  |
| Caregiver 3-B; Father, Rural | I try to make him play with kids, but when I see that they talk and play together but he can’t do that, and I sometimes feel bad when they neglect him. When I feel that way, I take him out and try to have him play with leaves… |  |  |
| Caregiver 4-B; Mother, Rural | There is a problem. I don’t like it when people pity her. I say to them: ‘what is wrong with her?’ and I even try to fight with them. On the taxi and on the street when people stare at her I say: ‘what are you looking at?’ May God give them understanding… |  |  |
| Caregiver 5-B; Mother, Rural | …the children in the village fear her they will say here she comes and run away.  …the children also fear her. They will run away saying ‘here she comes’. Deep down in my heart I always say how can I improve her [condition] may Allah do that I pray saying ‘you are the only one who have the ability I don't have the ability’… |  |  |
| Caregiver 6-B; Mother, Rural |  |  |  |
| Caregiver 7-B; Mother, Rural |  | …because my child is like this my husband was even asking me to leave. We were about to divorce. He was even saying: ‘I don’t want to see your eyes take your child and leave’. I was not telling this to my family. I was just crying… | It is very difficult. May God help us. If you have such kind of child, you need to be very strong…it is only recently that I started to mix with people…. up until he is 6 years old, he pees and poops on himself. I did not mix with people thinking that they may be disgusted. I felt like all people walking on the street are only looking at me…. |
| Caregiver 8-B; Mother, Rural | For him no to mix up with other children, there are some foolish people who think that his condition is contagious. We feel sorry inside. We would be happy if he could mix up and play with other children. He doesn’t hit people, even if they hit him, he doesn’t hit back, um, um, he doesn’t hit, in fact kids younger than him hit him… | Some people say that my child’s problem came because I have laughed [at other people] … it’s not that I have laughed, only Allah knows the reason why it came. |  |
| Caregiver 9-B; Father, Rural |  | I: …how did the other family members respond when they came to know about your child’s developmental disorder?  P: They told us to come clean if we have generational curse. I have noticed that people’s awareness around this issue is very poor.  I: Are you referring to people living around here?  P: People living around and, families, my sister-in-law told me to look for it in my family and she said that is why the child is not having any teeth or why he is not speaking. I told her that my family doesn’t have such things, we don’t have any of these things. I have other children and they are all fine except this child. So, I have nothing to look for in my family is what I told her. I hate my sister in law still now because she said those things... |  |

**Theme 2 & 3: Perceived impact of stigma and Parents’ positive social experiences/or lack of stigma**

| **Participants** | **Theme 2**  **Perceived impact of stigma** | **Theme 3**  **Parents’ positive social experiences/or lack of stigma** |
| --- | --- | --- |
| Caregiver 1-A |  |  |
| Caregiver 2-A |  | We got into a bus once and he [my child] snatched a hat from an older person, this person did not know about my son's condition. When my son took his hat, that person took the hat back and hit him. Another person, I think he knows about such children, asked that older person if it was appropriate to do that [hit the child] and they got into an altercation…. |
| Caregiver 3-A |  | …there is one home among our neighbours which he likes. He is playing with them and he likes them. He plays with them a lot. They look after him a lot. And if he [my child] likes you once, he always wants to be with you. They like him a lot and they give him food etc…  I: How is the attitude of people in other places?  P. One day when we go to Yekatit Hospital when he [my child with DD] was wrestling, one person warned me not to go with my child on a taxi next time.  I. Who was that person?  P. He was the driver assistant and the person sitting next to me asked me [about my child] and I told him that he has a problem. Then, he was about to fight with the assistant saying that he has a problem, he has autism… |
| Caregiver 4-A |  |  |
| Caregiver 5-A |  |  |
| Caregiver 6-A |  |  |
| Caregiver 7-A |  |  |
| Caregiver 8-A |  |  |
| Caregiver 9-A |  | when television is on at my friend’s house, to your surprise my daughter will tell you what is going to come next on the TV. This gives me hope, you understand what I mean. She will tell you the order, what comes at what time etc. when I look at my watch it is exactly as she said. To your surprise, she also says words that are difficult to say even for us. The words that she gets from the TV. I don’t have a TV at home, so I take her to my friend’s house and when she wants to sleep, I bring her back home.  I: Does your friend understand your child’s condition?  R: Yes, she is a mother. She is good, thank God. Since she is also related to my husband. Since she is related to us… |
| Caregiver 1-B | …the thing that I always ask myself is what is the effect of the community’s attitude on the child later. I swear to Allah I am very worried. I am worried that this may lead him to become mad…  …if we are three, me, my older one and my youngest, the older one gets uncomfortable because they [people] stare at his youngest brother, he asks me why they stare, and he gets upset. I tell him to ignore them…But I feel it inside me, I can imagine how he is feeling…  …truly, people are killing what is left of my energy. They are just making me be ashamed when I decide to take him [my child] out… I am starting to believe that in rural areas the mothers who hide their children behind closed doors are right. That is because this [mothers hiding their children] is a result of the public’s responses. Tomorrow, when I take my child out what could happen, will be worse… |  |
| Caregiver 2-B | …What I am worried a lot about is who is going to look after my child like I do if I die . Nobody will look after him. People are avoiding him even when I am here. If I die nobody will care about him… | Explaining the support that she received from a schoolteacher, one of the mothers noted that she took her child with DD to school when he was four-year-old (he is now nine-year-old). But none of the three schools she went to would accept him saying that his condition is beyond their capacity and that he needs special education. She recalled:  He [one of the teachers] calmed me down and encouraged me saying this kind of children need patience and it is not a curse…  If people know they would not say to me such [negative] things. People who know say ‘leave him alone’. People who have awareness understand when he exhibits difficult behaviour on the street…but most people do not understand and that is why I don’t take him to social places. |
| Caregiver 3-B |  |  |
| Caregiver 4-B | It is people, their attitude, which make you sick when you try to take your child out. This is maybe because of lack of awareness but their attitude is not good.  It is very difficult. When you take your child to the church you feel embarrassed. I used to think as if I am different. I used to say: ‘what have I done wrong’… | Thank God, she [daughter with DD] has increased love among us [me and my husband]. What I see in some families is rift [between husband and wife]. But here the love she has for her father is special. He is also good. Thank God. God has not disappointed me in this regard. When he gives me a child like her, he gave me a good husband… my family supports me. My mother lives a bit far from here, but she supports me morally. When I take my child to her place, I feel uncomfortable thinking that people may see her. My mother and sisters are not like that. They take her out… |
| Caregiver 5-B |  |  |
| Caregiver 6-B |  | I have not experienced stigma so far. People help me. I have not experienced such a thing… [for example] when I take my child to Black Lion Hospital people let me get into the taxi without queuing when they see my child biting me |
| Caregiver 7-B |  |  |
| Caregiver 8-B |  |  |
| Caregiver 9-B |  |  |

**Theme 4: Factors influencing affiliate stigma**

| **Participants** | **Factors influencing affiliate stigma** | |
| --- | --- | --- |
|  | Perceived family support and acceptance | Increased awareness about DD |
| Caregiver 1-A |  |  |
| Caregiver 2-A |  | I don't feel anything when people insult him [call him names] I used to cry before. There was a time I used to cry when people looked at my son asking why they were looking at him. We have learned about it here. We came here to make a change… |
| Caregiver 3-A |  |  |
| Caregiver 4-A |  |  |
| Caregiver 5-A | My family members have a good understanding of my child’s problem due to this they are giving him good love and care more than me. So, my family believe this and accepted me well.  As you know the thing about our community is, they talk behind your back when you go out with the child or when the child does something. But that does not stop me. I don’t feel ashamed. I go with him to the shop. I go with him to the church. I take him everywhere I want. People can say different things, but I don’t give much attention to that. |  |
| Caregiver 6-A |  |  |
| Caregiver 7-A |  | I: Tell me about the benefit of this training for you. For example, what changes have come because you took the training?  P. My neighbours may say many things, but I didn’t get upset and cry like the previous times because I took the training.  I. What did they say, for example?  P. as you know, people say many things. They consider it as a curse, and they say many other things and give many suggestions. Due to this, I cried many times before I took this training but now, I didn’t cry after I took this training. I didn’t say anything when they say different things and I prefer to be silent. This is the result of the training. |
| Caregiver 8-A |  |  |
| Caregiver 9-A | There is a huge pressure. There is exclusion starting from my family and there are very difficult situations, but I don’t have a choice but to accept that. There is a problem when we go on the road and when people see us and even in our family. You will be excluded eh... especially from my family, nobody accepted me including my mother. Because of this, I am not living in the community. I am living only with my child… |  |
| Caregiver 1-B |  | The quote below from one of the mothers in Butajira indicates that the information about DD that she gained from social media helped her to make sense of her mother’s stigmatising actions:  …my mother, I have told you, she used to say to take him [my child] to the back of the house when someone comes to the house…From what I have observed so far and what I have heard, from what I have seen from Facebook and the like, what boosted my morale is she does this out of fear [of embarrassment] and out of what the community gives back to her… |
| Caregiver 2-B | In my thinking it is Allah who gave me this child if people laugh let them laugh, I do not hide my child. I used to wash him, change his cloth and stand outside. When he runs, I follow him…people have improved their attitude now. It is better than earlier…the attitude of my neighbours [towards him] has improved now. They call his name and greet him. I see them having guilt regarding what they said about him….  He [my husband] looks after them [our children] if I want to go somewhere. He will be at home even if he has somewhere to go because there is nothing more important than x [our child]. He gives this much attention to his chid. There are fathers that do not give attention. There was this mother who come to PST carrying a baby who is not even three months old…I asked her why the father don’t come [to PST instead of her] she says he does not understand… | In the quote below, a mother talked about how parents should not bow to people’s negative attitudes and responses and hide their child, suggesting that her perception has changed since she started attending the CST intervention:  Parents of children with a developmental disorder should not hide their children. There are those who hide their children thinking that it is a curse. I am very sure there are lots of children who are hidden away. We [parents who attended CST] took out our children but I am very sure there are still many children who are hidden away. I say this because I know such children…in my thinking, a mother who carried her child for nine months should not feel ashamed of her child. She should take him out. If they say he is a sick person let them say that. People talk…they consider it as a curse, as an insult, and as something you bring because of witchcraft. But this is not the case…mothers should not be ashamed… |
| Caregiver 3-B |  |  |
| Caregiver 4-B | It is very difficult. You would take your child to the church and you would feel embarrassed. I used to think as if I am different. I used to say: ‘what have I done wrong?’. In this regard, I want to thank my family and my husband very much. They say: ‘you have not done anything wrong. you have been told; you know it’… | When I first knew about my child’s condition, I used to not talk about it. When neighbours asked me about my child, I used to say she has a heart problem and did not say she has a developmental problem. I considered it as something bad happened to me. I did not talk about it. It is only recently that I started to talk about it… after I became aware, I started to talk with confidence.  When I take her [my daughter] outside I used to feel uncomfortable. Her father is good. He takes her with him. But I did not have this kind of confidence. But now it [CST] has increased my confidence. |
| Caregiver 5-B |  |  |
| Caregiver 6-B |  |  |
| Caregiver 7-B | I don’t have anybody who helps me except God. It is me who does everything even my husband does not support me [crying]…. I feel very sorry for him [my child] when I see him being inferior to his friends [other children]. I say: what have I done wrong to God? [crying]…  It is very difficult. May God help us. If you have such kind of child, you need to be very strong…it is only recently that I started to mix with people…. up until he is 6 years old, he pees and poos on himself. I did not mix with people thinking that they may be disgusted. I felt like all people walking on the street are only looking at me …. |  |
| Caregiver 8-B |  |  |
| Caregiver 9-B |  |  |

**Appendix B: Topic guides**

1. **Topic guide for interview with caregivers in Addis Ababa post CST pre-pilot testing**

Family ID_______________ Participant ID: ____________________

Family Member:

 mother  father

 grandmother  grandfather

 sister/stepsister  brother/stepbrother

 other (specify) __________________________

Date: ___________________

Location: ________________

A. **Introduction**

Interviewer to say his/her name and where they are from.

We are here to learn more about your experiences taking part in skills training for caregivers of a child who develops slowly. The information you give us will help us to further adapt the training as necessary, so that it meets the needs of families in Ethiopia. While we are talking, we are recording what is said with this tape recorder so that we can remember all of the information. Everything we learn from people will be put together so that we have an idea of the views and experiences of many different families. There are no right or wrong answers.

B. **Interview guide**

[Start tape recording from here, start tape recording with saying the family ID number, but not the name of the caregiver]

- Ask about experiences with programme, both positive and negative.

Please tell me about your experience of the programme

What did you like about the programme?

What didn’t you like about the programme?

- Explore the usefulness of the programme to the caregivers.

Tell me about how relevant the programme was to your circumstances.

How useful was the programme? In what ways was it useful?

Did the programme help you to feel more confident? In what ways?

Did the programme help you to feel less stressed? In what ways?

Did the programme help you to improve your child’s skills? In what ways?

Was the programme useful in ways that you did not foresee or expect? Please tell me in what ways?

- Explore usefulness of CST components and strategies.

What part of the programme was most useful to you? Why?

Tell me about your experience of the home visits? What were the positive and the negative aspects?

How did you find the videoing?

Tell me about your experience of group sessions? What were the positive and the negative aspects?

Which strategy that you learned was the most useful? Why?

- Ask for suggestions to improve relevance and usefulness of CST content.

What else would you have liked the CST programme to address?

What other important messages do you think need to be conveyed to caregivers of children with developmental delays or developmental disorders?

- Explore acceptability of programme content.

How did you find the messages and stories that were used in the CST sessions? How much did those messages and stories fit with your situation?

- Ask about experience and difficulties with implementing the strategies at home and suggestions to make homework more feasible.

What made it hard to implement the intervention with your family? What helped to make it easier?

- Explore barriers with attending the programme and suggestions to improve attendance and

reduce drop out.

What made it easy/hard for you to attend the training sessions? Any suggestion to make it easier to attend?

- Explore programme impact on families.

What kind of impact has the training had on you? How about on your child? How about other

members of your family? How about on relationship among family members

- Explore if the programme implementation has triggered any change in the community.

How do you find getting out and about in your community? Has the programme affected that in any way? Please tell me about that. [Probe about awareness, accessing support, stigma]

- Explore adverse effect on caregivers, families and communities.

Did you or your family experience any difficulty or challenge or discomfort in relation to attending the CST programme?

Have you experienced any negativity from your community since the start of the CST programme implementation? [If so, ask for suggestions to avoid this in the future.]

- Ask for any additional suggestions and thank participants.

Is there anything else you would change about the programme?

Are there any other thoughts or comments you would like to share with us?

**ID code □□□□□**

**2. Topic guide for interview with caregivers of a child with a developmental disorder in Butajira**

**A. Introduction**

Interviewer to say his/her name and where they are from.

We are here to learn about your thoughts and experiences regarding raising a child with a developmental disorder (a child who develops slowly). While we are talking, we are recording what is said with this tape recorder so that we can remember all the information. Everything we learn from people will be put together so that we have an idea of the views and experiences of many different families. There are no right or wrong answers.

**B. Background**

| S. N | First of all, we just need some background information. | | | |
| --- | --- | --- | --- | --- |
| 1 | What is your gender? | Male | 1 | GEND |
|  |  | Female | 2 |  |
| 2 | What age are you now? | _________ years | | AGE |
|  | Date of Birth (if known) | [ ] | |  |
|  | Today’s date | [ ] | |  |
| 3 | How much formal education have you received? | No formal education [ ] | | EDU |
|  |  | Completed grade [ ] [ ] | |  |
| 4 | What is your marital status? | Married | 1 | MARSTAT |
|  |  | Single | 2 |  |
|  |  | Divorced | 3 |  |
|  |  | Widowed | 4 |  |
| 5 | Do you practise a religion? |  |  | RELIG |
|  |  | Orthodox Christian | 1 |  |
|  |  | Protestant | 2 |  |
|  |  | Catholic | 3 |  |
|  |  | Muslim | 4 |  |
|  |  | Other (please specify): __________ | 66 |  |
| 6 | What is your occupation? | Housewife | 1 | OCCU |
|  |  | Student | 2 |  |
|  |  | Unemployed | 3 |  |
|  |  | Farmer | 4 |  |
|  |  | Government employee | 5 |  |
|  |  | Private employee | 6 |  |
|  |  | Trader | 7 |  |
|  |  | Other (please specify):______________ | 66 |  |
| 7 | What is your relationship to the child who has problems with development we will be talking about today? | Mother | 1 | RSWCDD |
|  |  | Father | 2 |  |
|  |  | Brother | 3 |  |
|  |  | Sister | 4 |  |
|  |  | Grandmother | 5 |  |
|  |  | Grandfather | 6 |  |
|  |  | Aunt | 7 |  |
|  |  | Uncle | 8 |  |
|  |  | Other (please specify): ____________________________ | 66 |  |
| 8 | Do you live with the child? | Yes | 1 |  |
|  |  | NO | 2 |  |
| 9 | How old is the child with problems with development? | ____________________________ |  |  |
| 10 | Is this child a boy or a girl? | Boy | 1 | GEND |
|  |  | Girl | 2 |  |
| 11 | How many children do you have? |  |  | NOC |
| 12 | Do any of your other children have developmental problems? | Yes | 1 | DP |
|  |  | NO | 2 |  |

If yes, please give details about their problems:

**C. Interview guide**

**[Start tape recording from here, start tape recording with saying the participant ID number, but not the name of the caregiver]**

**[Use Prompts only when a respondent does not readily answer questions]**

- When did you recognize that your child has a developmental disorder? (When did you recognize that your child is developing slowly?)
- What did you do when you came to know that your child is developing slowly?

Prompts:

- Did you seek medical help?
- Did you get traditional treatment (e.g. going to holy water place)?
- How did your family respond to learning that your child has a developmental disorder?
- What are the challenges of raising a child with developmental disorder?

Prompts:

- Managing child’s difficult behaviour
- Lack of understanding from others (e.g. when out in public places)
- Social isolation
- Financial burden
- Difficulty getting appropriate health service
- Difficulty getting appropriate education
- How do you deal with these challenges?
- What is helping you to deal with these challenges?

Prompts:

- Having a supportive/understanding family, relatives, friends and neighbours?
- Religion?
- Has having a child with DD changed your family’s life? If so, how?

Prompts:

Social:

- In terms of relationship among family members (with husband/wife, with siblings, among siblings)?
- In terms of relationship with neighbours?
- In terms of relationship with relatives?
- In terms of relationship with friends?

Psychological:

- Increased stress?
- Do you feel like your relationship with other people has changed because of having a child with DD? If so, how?
- Do you include your child with DD in family events and gatherings? If so, how? How do other family members react? If no, why?
- Do you feel that people treat you or your family differently because of your child's developmental disorder?

If yes, can you please elaborate on the nature of other people’s reactions?
